# An Active and Soft Hydrogel Actuator to Stimulate Live Cell Clusters by Self-folding

**DOI:** 10.3390/polym12030583

**Published:** 2020-03-05

**Authors:** Jun Woo Lim, Hee-jin Kim, Yechan Kim, Sung Gyu Shin, Sungwoo Cho, Woong Gyu Jung, Jae Hyun Jeong

**Affiliations:** 1 Department of Chemical Engineering, Soongsil University, Seoul 06978, Korea; ljw9424@soongsil.ac.kr (J.W.L.); whitegd45@ssu.ac.kr (S.G.S.); som113@ssu.ac.kr (S.C.); 2 School of Chemical and Biological Engineering, Seoul National University, Seoul 08826, Korea; kimyechan94@snu.ac.kr; 3 Department of Biomedical Engineering, Ulsan National Institute of Science and Technology (UNIST), Ulsan 44919, Korea; wgjung@unist.ac.kr

**Keywords:** hydrogel actuator, self-folding, expansion ratio, live cell clusters

## Abstract

The hydrogels are widely used in various applications, and their successful uses depend on controlling the mechanical properties. In this study, we present an advanced strategy to develop hydrogel actuator designed to stimulate live cell clusters by self-folding. The hydrogel actuator consisting of two layers with different expansion ratios were fabricated to have various curvatures in self-folding. The expansion ratio of the hydrogel tuned with the molecular weight and concentration of gel-forming polymers, and temperature-sensitive molecules in a controlled manner. As a result, the hydrogel actuator could stimulate live cell clusters by compression and tension repeatedly, in response to temperature. The cell clusters were compressed in the 0.7-fold decreases of the radius of curvature with 1.0 mm in room temperature, as compared to that of 1.4 mm in 37 °C. Interestingly, the vascular endothelial growth factor (VEGF) and insulin-like growth factor-binding protein-2 (IGFBP-2) in MCF-7 tumor cells exposed by mechanical stimulation was expressed more than in those without stimulation. Overall, this new strategy to prepare the active and soft hydrogel actuator would be actively used in tissue engineering, drug delivery, and micro-scale actuators.

## 1. Introduction

Mechanical stiffness of extracellular matrix (ECM) has been proven in many studies to act as insoluble signal to regulate diverse cellular phenotypic activities, including proliferation, differentiation, and gene expression [1,2,3]. In addition, compressive stress also modulates various phenotypic activities of cells, such as proliferation, differentiation, apoptosis and even metastasis [4,5,6,7]. However, there are few successes in understating the role of compressive stress of cells mechanistically, because of limited technologies to apply the mechanical stress upon live cells without damages, over physiologically relevant length scales. Therefore, this study presents a novel strategy to develop a soft hydrogel actuator designed to stimulate live cell clusters by self-folding. Generally, the hydrogels form a three-dimensional network structure having a bunch of water which is similar to that of ECM [8]. In addition, the physical properties of the hydrogels including stiffness and permeability can be controlled by altering elastic modulus and swelling ratio in a desired manner. Recently, the multi-layered hydrogels which have different physical and chemical properties of each layer have been suggested as a method to mimic anisotropic and complex structures of tissues [9]. Multi-layered hydrogel with different swelling ratio of each layer can be fabricated to have various curvatures when exposed to aqueous media. By adding a sensitive material to temperature, pH, and light, it is possible to design a multi-layered hydrogel that transforms to have various curvatures, in response to conditions [10,11,12,13].

**Figure 1:**
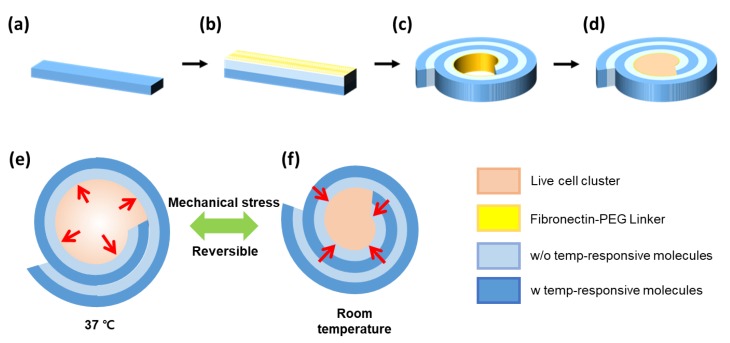
A schematic diagram of each step for preparing the active hydrogel actuator to stimulate with bi-layered hydrogel. The hydrogel actuator was assembled by preparing 1st thin layer (**a**), and subsequently, 2nd layer was prepared over the 1st layer (**b**). The subsequent immersion of the prepared bi-layered hydrogel in an aqueous solution triggered self-folding with designated curvatures (**c**). Finally, a live cell cluster was introduced inside of the self-folded hydrogel (**d**) and stimulated by compression and tension repeatedly, in response to temperature (**e**,**f**).

Therefore, we hypothesized that a hydrogel consisting of two layers with different capacities to uptake water would be a soft actuator to stimulate live cells by self-folding and unfolding, in environment conditions. In this study, poly (ethylene glycol) diacrylate (PEGDA) was used as a gel-forming polymer. The expansion ratio of a hydrogel was tuned with the molecular weight and concentration of PEGDAs, and temperature-sensitive molecules in a controlled manner. First, a hydrogel was formed by photo-crosslinking, consisting of two layers. One layer was crosslinked with introduced temperature-sensitive molecules, but the other one has no temperature-sensitive molecules (Figure 1a). Then, the surface of bi-layered hydrogel was treated with cell adhesion proteins to allow cell clusters to adhere (Figure 1b–d). Mechanical stimulation was performed by introducing a cell cluster into the prepared bi-layered hydrogel (Figure 1e,f). Finally, the bi-layered hydrogel acting as an actuator could stimulate live cell clusters by compression and tension repeatedly, in response to temperature. In the results, the cell clusters were compressed in the 0.7-fold decreases of the radius of curvature with 1.0 mm in room temperature, as compared to that of 1.4 mm in 37 °C. Interestingly, the VEGF and IGFBP-2 in MCF-7 tumor cells exposed by mechanical stimulation were expressed more than in those without stimulation. This new strategy to prepare the active and soft hydrogel actuator would be actively used as micro-scale and soft actuator, and further regulate and evaluate the effect of compressive stress on the live cells and tissue. 

## 2. Materials and Methods 

### 2.1. Hydrogel Preparation

Hydrogels were formed with poly (ethylene glycol) diacrylate (PEGDA) with PEGDA-3400 (M_n_ of 3400 g/mol, Alfa Aesar, Haverhill, MA, USA) and PEGDA-575 (M_n_ of 575 g/mol, Sigma, St. Louis, MI, USA) by photo-crosslinking. First, pre-gelled solutions were prepared by mixing 12~20 wt% PEGDA in deionized (DI) water to determine the expansion ratio of a formed hydrogel with concentration. Then, N-isopropylacrylamide (NIPAM, Sigma, St. Louis, MI, USA) was introduced to the pre-gelled solution in order to evaluate the temperature-sensitivity of a hydrogel in terms of degree of swelling. The temperature-sensitive PEGDA hydrogel was prepared with a concentration of 0–6 wt% NIPAM and 20–14% PEGDA-3400. The photo-initiator, 2-hydroxy-40-(2-hydroxyethoxy)-2-methylpropiophenone (Irgacure-2959, Sigma, St. Louis, MI, USA) was dissolved in dimethyl sulfoxide (DMSO, Sigma, St. Louis, MI, USA) at 10 wt% stock solution and added to the pre-gelled solution to form 0.2 wt% as the final concentration. Then, the prepared pre-gelled solution was cast between quartz plates with 1 mm of thickness as a spacer. Finally, the hydrogel was formed by exposing the cast pre-gelled solution to ultraviolet (UV) lamp (365 nm, VL-4.LC, VILBER LOURMAT, Collégien, France) for gelation about 10 min. Then, the prepared gel was punched out in a form of a disk with a diameter of 8 mm. The gel disks were incubated in DI water until the gel was fully swelled in saturation, before characterization.

### 2.2. Characterizations of a Formed Hydrogel

The swelling ratio (*Q_m_*), the expansion ratio (*S*), and the elastic modulus (*E*) of each layer were measured to fabricate the hydrogel actuator in a desired manner. 

The weight of hydrogel (*W_s_*) was measured after incubation in DI water over 12 h. The weight of hydrogel (*W_d_*) was measured after drying at 60 °C over 12 h. The swelling ratio was calculated using the Equation (1).

(1) $$Q_{m}=\left(\frac{W_{s}-W_{d}}{W_{d}}\right)\times 100$$

The expansion ratio was characterized with swelling ratio of hydrogel when prepared (*Q_i_*) and after (*Q_f_*) fully incubation in DI water. When the bi-layered hydrogel is prepared, both layers are prepared to the same specification in the bi-layered system, so the *Q_i_* of the initially prepared hydrogel should be measured. The expansion ratio was defined as the one-dimensional expansion of the hydrogel and calculated using the Equation (2).

(2) $$S={\left(\frac{Q_{f}}{Q_{i}}\right)}^{\frac{1}{3}}-1$$

The elastic modulus of the hydrogels was measured using a universal testing machine (UTM, DrTech, Seongnam-si, Korea). The hydrogels were compressed at 10% strain and a constant rate of 1.0 mm/min, a load range of 1.0 kg·f after incubation over 24 h. 

A bimetallic strip curvature equation was adopted to predict the self-folding of the prepared bi-layered hydrogel. A bimetallic strip curvature is defined in Equation (3),

(3) $$r=\frac{E_{1}{}^{2}t_{1}{}^{4}+4E_{1}E_{2}t_{1}{}^{3}t_{2}+6E_{1}E_{2}t_{1}{}^{2}t_{2}{}^{2}+4E_{1}E_{2}t_{1}t_{2}{}^{3}+E_{2}{}^{2}t_{2}{}^{4}}{6E_{1}E_{2}\left(t_{1}+t_{2}\right)t_{1}t_{2}\Delta \varepsilon }$$

where *E*_1_, *E*_2_ was elastic modulus and *t*_1_, *t*_2_ was thickness of each layer, ∆*ε* was difference of expansion ratio of each layer [14,15].

### 2.3. Fabrication of Hydrogel Actuator Consisting of Two Layers

The hydrogel actuator was composed of bi-layered hydrogel varying of different composition, in a form of a strip with 10 mm in length and 1 mm in width. For example, the bi-layered hydrogel was assembled by first preparing a hydrogel with concentration of 14 wt% PEGDA-3400 and 6 wt% NIPAM, by exposing the pre-gel solution to UV light. Subsequently, second hydrogel layer was prepared over the first hydrogel layer by introducing the pre-gelled solution of solely 20 wt% of PEGDA-575. Each layer was prepared with 0.2 wt% of Irgacure2959 in pre-gelled solution. Finally, the top surface of the second layer was micro-patterned with fibronectin (Fn, from human plasma, Sigma, St. Louis, MI, USA) by μCP (micro-contact printing) method, in order to attach live cell clusters and not to be detached in enlarged radius of curvature in stimulation process. Fn-linker solution was prepared by adding poly(ethylene glycol) linker (PEG linker) solution (Acrylate PEG NHS Ester, 3500 g/mol, JenKem Technology, Plano, TX, USA, 5.0 mg/mL) to 0.2 mg/mL of fibronectin. The prepared Fn-linker solution was placed on top of a polydimethylsiloxane (PDMS, Dow Corning, Midland, MI, USA) stamp having the same size as the surface of the hydrogel actuator at 37 °C for 2 h, and the remaining solution was removed after 2 h. Then, the excess Fn-linker solution was removed, and the PDMS stamp was dried by blowing dry N_2_ gas. The PDMS stamp was placed on top of a quartz plate and gently pressed, and then incubated at 37 °C for 1 h. After the Fn-linker was printed, the hydrogel actuator was fabricated by UV irradiation (365 nm) on top of a printed quartz plate. To confirm the covalent attachment of fibronectin on the hydrogel surface, the fibronectin-conjugated hydrogels were incubated overnight at room temperature in anti-fibronectin antibody produced in mouse (Sigma, St. Louis, MI, USA) solution diluted in phosphate-buffered saline (PBS, Biowest, Nuaillé, France) PBS at 1:500. After the gels were rinsed three times with PBS, the gels were incubated for 2 h at 37 °C in Alexa Fluor 488 goat anti-mouse IgG (Abcam, Cambridge, UK) solution diluted in PBS at 1:500. After another rinsing three times with PBS, the fluorescent image of fibronectin on the hydrogel surface was confirmed with a fluorescence microscope (Nikon Eclipse Ti, Nikon, Tokyo, Japan) [16].

### 2.4. Preparation of Tumor Cell Clusters and Stimulation by the Hydrogel Actuator

Tumor cell clusters were prepared in a cylindrical form according to the dimension of the hydrogel actuator. A mold for cylindrical cell clusters was prepared using 1.0 wt% of agarose (Sigma, St. Louis, MI, USA) solution at 6-well plate and had holes with a diameter of 1.5 mm. MCF-7 cells (KCLB, Seoul, Korea) were cultured into a 75 cm^2^ flask using Roswell Park Memorial Institute medium (RPMI 1640, Biowest, Nuaillé, France) complete media supplemented with 10% of fetal bovine serum (FBS, Biowest, Nuaillé, France), 1.0% of Penicillin-Streptomycin (P/S, Biowest, Nuaillé, France) in a 5% CO_2_ incubator at 37 °C. The cultured MCF-7 cells were taken by treatment with trypsin, and 1 × 10^6^ cells were injected into the mold in a 5% CO_2_ incubator at 37 °C for 1 h. After 1 h, the culture media was put into 6-well plates and incubated for 24 h. The prepared cell clusters were introduced into the hydrogel actuator and cultured in culture medium to be attached to the Fn-linker treated surface of the hydrogel actuator for 12 h. The tumor cell clusters introduced into the hydrogel actuator were stimulated by compression and tension 4 times a day for 5 days. The angiogenesis factor of tumor cell clusters following mechanical stimulation was analyzed using angiogenesis array kit (Proteome ProfilerTM (R&D Systems, Inc., Minneapolis, MN, USA), according to the manufacturer’s instructions. The treated detection membrane was imaged using ChemiDoc^TM^ MP (BIO-RAD, Hercules, CA, USA). The pixel density of the membrane was analyzed using the Image*J*.

## 3. Results and Discussions

### 3.1. Characteriazetion of PEGDA Hydrogels

**Figure 2:**
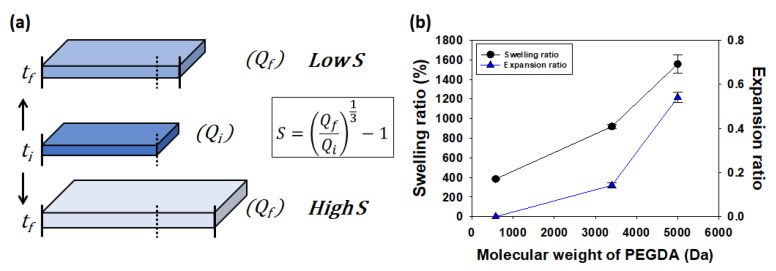
(**a**) A schematic diagram of expansion ratio which is determined with swelling ratio of a hydrogel when prepared (*Q_i_*) and after (*Q_f_*) fully incubation in aqueous media. *t*_i_ and *t*_f_ are time of initial state and final state of the hydrogel. (**b**) The swelling ratios (*Q*, left axis) and expansion ratios (*S*, right axis) were measured and calculated for 20% of PEGDA hydrogels as a function of MWs. Note that NIPAM was not included in these gels.

The bi-layered hydrogel was assembled by first preparing a thin hydrogel of PEGDA with low molecular weight, termed as PEGDA-575, by exposing the pre-gelled solution to UV. Subsequently, another hydrogel layer was prepared over the first layer of PEGDA-575 by crosslinking the higher molecular weight of PEGDA solution. Then, when the hydrogel was exposed to aqueous media, it triggered self-folding with decreasing radius of curvature. The radius of curvature is dependent on differences in the molecular weight and concentrations between PEGDA gel layers. Specifically, increasing the molecular of weight of PEGDAs resulted in an increase in the expansion ratio as defined in Equation (1) to predict the radius of curvature in the bi-layered hydrogel system (Figure 2b). It is noted that the swelling ratio refers the water content of the hydrogel against the dried mass, but the expansion ratio refers the difference in the swelling ratio (*Q_f_*) of hydrogel in the saturation state after incubation, and the swelling ratio (*Q_i_*) in the initial state right after prepared (Figure 2a) [17]. 

**Figure 3:**
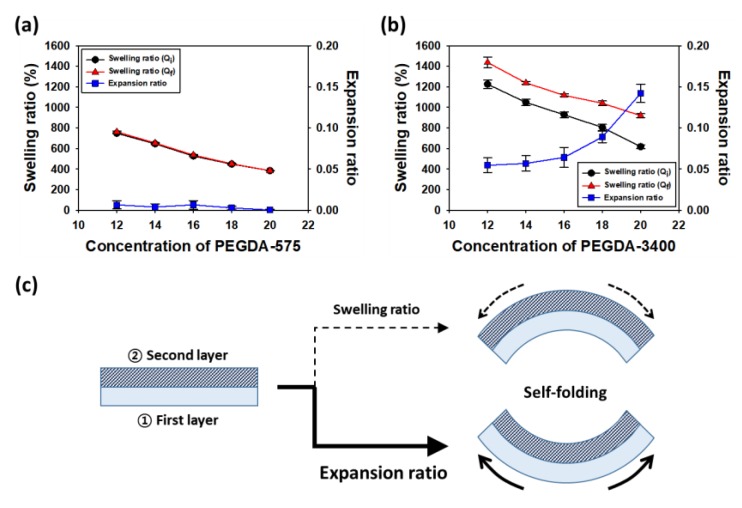
The swelling ratios and expansion ratios of the hydrogels prepared with varying of MWs and concentrations of PEGDA-575 (**a**) and PEGDA-3400 (**b**). Noted that NIPAM was not included in these gels. (**c**) The expansion ratios instead of the swelling ratios are used to design the self-folding hydrogel with designated curvature in a bi-layered hydrogel system.

The radius of curvature of the bi-layered hydrogel was further modulated with the polymer concentration by varying of the expansion ratio. As shown in Figure 2a, in the case of PEGDA-575 hydrogels, the swelling ratio in the saturation state (*Q_f_*) and initial state (*Q_i_*) showed no significant difference, as the concentration decreased. It means that the expansion ratio of the hydrogel formed with low molecular weight of PEGDAs has an almost value of zero, regardless of their concentration [18,19,20]. On the other hand, in the case of PEGDA-3400 hydrogels, the difference of swelling ratio before and after incubation decreased as the concentration decreased. Subsequently, the expansion ratio also decreased as the concentration decreased (Figure 3b) [21,22]. Interestingly, it was found that the expansion ratio decreased while the swelling ratio increased, as the concentration decreased. In particular, the swelling ratio increases as the concentration decreases while keeping the molecular weight of polymer, not because of the expansion ratio of a hydrogel, but because of simply reduced total polymer mass (*W*_d_) in the Equation (1). Therefore, the radius of curvature of the bi-layered hydrogel should be predicted and designed with the difference in the expansion ratio between two gel layers, instead of the value of swelling ratio. For example, when the bi-layered hydrogel is assembled by preparing of 1st layer of 20% and 2nd layer of 12% in PEGDA-3400, the hydrogel bents convex down as shown in Figure 3c. If one predicts the self-folding based on the swelling ratios (1000% of first layer and 1400% of second layer), it might be mistaken that the hydrogel bents convex up. 

### 3.2. The Self-folding of the Hydrogel Actuator and Stimulation of Live Cell Clusters

**Figure 4:**
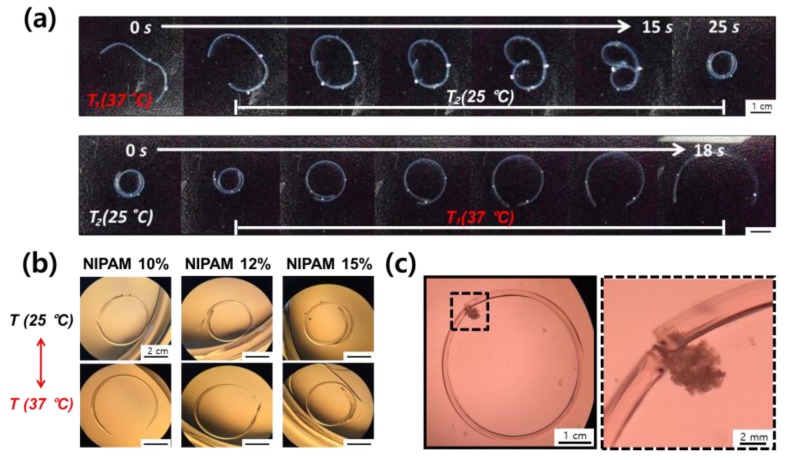
(**a**) The bi-layered hydrogel with temperature-sensitive molecules transforms by self-folding with various curvatures, in response to temperature. The time represents the process of self-folding and unfolding of the bi-layered hydrogel from 37 °C to 25 °C and vice versa, until it reached to equilibrium. (**b**) The bi-layered hydrogel with various curvatures controlled with degree of introduced temperature-sensitive molecules (**b**) grabs a live cell clusters (**c**).

The expansion ratio of a hydrogel was further tuned by introducing temperature-sensitive molecules, with the molecular weight and concentration of PEGDAs. First, a hydrogel was formed by photo-crosslinking, consisting of two layers. One layer was crosslinked with introduced temperature-sensitive molecules, but the other one has no temperature-sensitive molecules. Then, the bi-layered hydrogel with temperature-sensitive molecules transforms by self-folding with various curvatures, in response to temperature (Figure 4a). Even, the bi-layered hydrogel could be prepared to grab a live cell clusters by adjusting the radius of curvature (Figure 4b,c).

**Figure 5:**
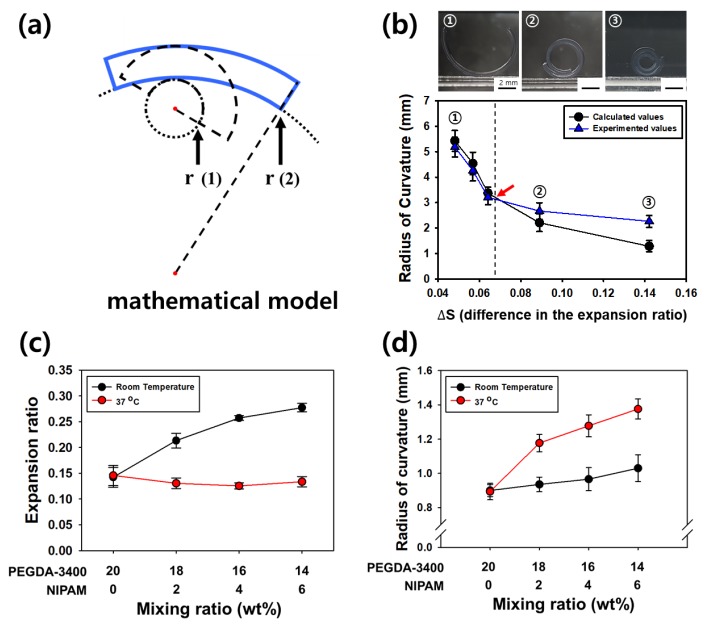
(**a**) Radius of curvatures of the bi-layered hydrogel estimated by a mathematical model developed for the curvature of a heat-induced bimetallic strip. (**b**) Theoretical and experimental radius of curvatures according to the difference in the expansion ratio of two layers. The arrow marks the radius of curvature in which the hydrogel bents one wheel. (**c**) Effects of PEGDA-3400 and NIPAM concentration on the expansion ratio of the hydrogel at room temperature and 37 °C. (**d**) Experimental radius of curvatures of thermos-sensitive bi-layered hydrogels.

Figure 5c show the effects of PEGDA-3400 and NIPAM concentration on the expansion ratio of the hydrogel at room temperature and 37 °C. The expansion ratio increased by increasing concentration of NIPAM at room temperature, while the total concentration of PEGDA-3400 and NIPAM is constant. Actually, PEGDA has two acrylate groups and they act as a crosslinking agent as well as a backbone polymer. In contrast, NIPAM has one acrylate group and forms p-NIPAM chain. The p-NIPAM chain is just linked to the PEGDA networks, leading to increase the expansion ratio of hydrogel. In addition, the expansion ratio of PEGDA-3400-NIPAM hydrogel was almost constant with increasing concentration of NIPAM at 37 °C above the lower critical solution temperature (LCST) of NIPAM [23]. Figure 5a shows the schematics of the radius of curvature from the hydrogel of each layer characteristics. The radius of curvature of the bi-layered hydrogel can be evaluated using the bimetallic Equation (3) with values of elastic modulus (*E*_1_, *E*_2_), thickness (*t*_1_, *t*_2_), and difference (Δε) in the expansion ratio of two layers. Actually, the equation fits well for the self-folding of hydrogel until where the gel bents one wheel, but after that, the theoretical and experimental values are quite different, as shown in Figure 5b. It is caused from the contact occurred in self-folded part when the hydrogel bents to overlapping. The contact creates a constraint which would alter the radius of curvature. Finally, the Figure 5d shows the experimental radius of curvatures in the bi-layered hydrogels in response to temperature. For example, when the bi-layered hydrogel was prepared with first layer consisting of 16 wt% of PEGDA-3400 and 4 wt% of NIPAM, and the second layer consisting of solely 20 wt% of PEGDA-575, the final hydrogel self-folded reversibly with 1.0 mm of radius of curvature at 25 °C and 1.4 mm of that at 37 °C. However, no difference in the curvature shows in hydrogel without NIPAMs, regardless of temperature. 

**Figure 6:**
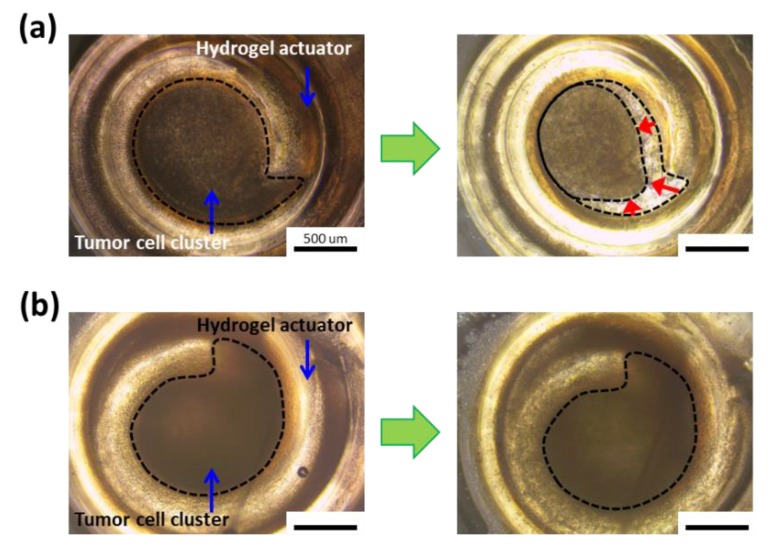
The hydrogel actuator without cell adhesion proteins gives only once compressive stress onto the cell clusters, because the non-adherent cell cluster caused a permanent compaction upon first compression by the actuator (**a**). However, the surface of bi-layered hydrogel was treated with cell adhesion proteins to allow cell clusters to adhere, and then adhered cell cluster can be stretched when the radius of curvature increases (**b**).

Finally, the bi-layered hydrogel acting as an actuator could stimulate live cell clusters by compression and tension repeatedly, in response to temperature. Note that the quantification of applied forces is beyond the scope of this paper, so that it would be examined and reported in another study. First, the tumor cell (MCF-7) clusters were prepared using a 1.0 wt% of agarose mold and introduced into the hydrogel actuator. The surface of bi-layered hydrogel was treated with cell adhesion proteins to allow cell clusters to adhere, and then adhered cell clusters can be stretched when the radius of curvature increases. Therefore, the bi-layered hydrogel could stimulate live cell clusters by repeated compression and tension (Figure 6b). In contrast, the hydrogel without cell adhesion proteins gives only once compressive stress onto the cell clusters at 25 °C, since the non-adherent cell cluster caused a permanent compaction upon the first compression by the actuator (Figure 6a). 

**Figure 7:**
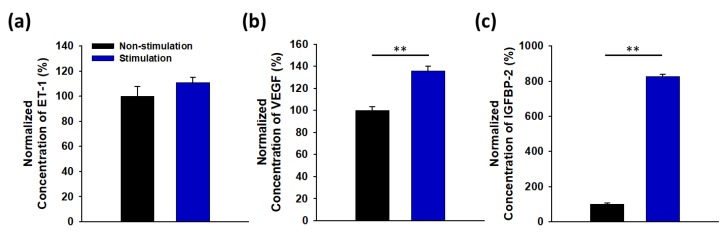
Comparison of expression levels of angiogenesis factors expressed from tumor cell clusters by stimulation. (**a**) Endothelin-1 (ET-1); (**b**) Vascular Endothelial Growth Factor (VEGF); (**c**) Insulin-like growth factor-binding protein-2 (IGFBP-2). (** *p* < 0.05).

Finally, the bi-layered hydrogel acting as an actuator could stimulate live cell clusters by compression and tension repeatedly, in response to temperature. Specifically, the tumor cell clusters were stimulated by compression and tension 4 times a day for 5 days. The cell clusters were compressed in the 0.7-fold decreases of the radius of curvature with 1.0 mm in room temp., as compared to that of 1.4 mm in 37 °C. The cells adhered into the hydrogel actuator exhibited no critical damage during stimulating, as confirmed with the larger intracellular cleavage of yellow tetrazolium salt (MTT) into a purple formazan product. The MCF-7 cell clusters are expressing multiple angiogenic factors, including VEGF (vascular endothelial growth factor), Endothelin-1 and IGFBP-2 (Insulin-like growth factor-binding protein-2). Interestingly, when the MCF-7 cells were exposed by squeezing and releasing, the cellular expression levels of VEGF and IGFBP-2 were higher than those without stimulation (Figure 7). This result indicates that the mechanical stress induced into tumor cells clusters would stimulate to express multiple angiogenic factors related on angiogenesis, and trigger on sprouting of neovessels into the tumor cells [24,25,26]. 

In summary, the hydrogel was formed by photo-crosslinking, consisting of two layers. One layer was crosslinked with introduced temperature-sensitive molecules, but the other one has no temperature-sensitive molecules. Then, the surface of bi-layered hydrogel was treated with cell adhesion proteins to allow cell clusters to adhere. Mechanical stimulation was performed by introducing a cell cluster into the prepared bi-layered hydrogel. Finally, the bi-layered hydrogel acting as an actuator could stimulate live cell clusters by compression and tension repeatedly, in response to temperature. Interestingly, the VEGF and IGFBP-2 in MCF-7 tumor cells exposed by mechanical stimulation were expressed more than in those without stimulation. We believe that the developed hydrogel actuator would be actively used to further regulate and evaluate the effect of compressive stress on the live cells and tissue.

## 4. Conclusions

Overall, this study presents an active and soft hydrogel actuator using bi-layered hydrogel to stimulate live cell clusters by self-folding. The expansion ratio of the hydrogel was tuned with the molecular weight and concentration of gel-forming polymers, and temperature-sensitive molecules in a controlled manner. In addition, the curvature induced by the difference of their expansion ratio could be controlled with introducing the temperature-sensitive molecules. As a result, the hydrogel actuator could stimulate live cell clusters by compression and tension repeatedly, in response to temperature. In the results, the cell clusters were compressed in the 0.7-fold decreases of the radius of curvature with 1.0 mm in room temp., as compared to that of 1.4 mm in 37 °C. Interestingly, the VEGF and IGFBP-2 in MCF-7 tumor cells exposed by mechanical stimulation was expressed more than in those without stimulation. Overall, this new strategy to prepare the active and soft hydrogel actuator would be actively used in tissue engineering, drug delivery, and micro-scale actuator. Specifically, developed hydrogel actuator would be used to further regulate and evaluate the effect of compressive stress on the live cells and tissue.
